# Unethical pro-organizational behavior and actors’ work attitudes, behaviors, and performance: a meta-analysis

**DOI:** 10.3389/fpsyg.2025.1697571

**Published:** 2026-01-27

**Authors:** Zhuojie Li, Xiaozhan Wang, Paweł Jurek, Leiru Wei

**Affiliations:** 1 Institute of Psychology, Faculty of Social Sciences, University of Gdansk, Gdansk, Poland; 2 College of Economics and Management, Zhengzhou University of Light Industry, Zhengzhou, China

**Keywords:** deviant workplace behavior, meta-analysis, organizational citizenship behavior, unethical pro-organizational behavior, work attitude, work performance

## Abstract

**Introduction:**

Unethical Pro-Organizational Behavior (UPB), as a special extra-role behavior with both pro-organizational and unethical characteristics, its impact on actors’ work outcomes has not yet reached a consensus. To address this gap, this study adopts a meta-analytic approach.

**Methods:**

Based on Affective Events Theory, this study conducts a meta-analysis of 44 literature (including 55 independent studies, 182 effect sizes, and 18,074 research samples) to systematically integrate the relationships and boundary conditions between UPB and actors’ work attitudes, work behaviors, and work performance, and explore the mediating role of emotions.

**Results:**

The results show that: (1) UPB is significantly positively correlated with negative work attitudes (r̄ = 0.242, 95% CI = [0.098, 0.376]), organizational citizenship behavior (OCB) (r̄ = 0.226, 95% CI = [0.070, 0.372]), and deviant workplace Behavior (DWB) (r̄ = 0.232, 95% CI = [0.127, 0.331]), but not significantly correlated with positive work attitudes (r̄ = − 0.078, 95% CI = [−0.388, 0.248]) and work performance (r̄ = 0.125, 95% CI = [−0.049, 0.292]). (2) Boundary condition tests show that sampling methods (multi-wave vs. cross-sectional), cultural backgrounds (Eastern vs. Western) and gender have significant moderating effects on the above relationships. Specifically, multi-wave sampling strengthens the association between UPB and negative work attitudes; under Eastern cultural backgrounds, the impact of UPB on OCB and DWB is stronger; the higher the proportion of females, the weaker the association between UPB and DWB. (3) Mediating mechanism analysis reveals that UPB indirectly reduces negative work attitudes and indirectly promotes OCB through positive emotions. However, the mediating role of positive emotions in the relationship between UPB and DWB is not significant. Additionally, it indirectly promotes negative work attitudes through negative emotions, while the mediating role of negative emotions in the relationships between UPB and both OCB and DWB are not significant.

**Discussion:**

The conclusions of this study reveal the complexity of UPB’s impact and the key mediating role of emotions, providing theoretical basis and practical enlightenment for organizations to manage employees’ UPB.

## 1 Introduction

As the external environment of enterprises becomes increasingly complex and dynamic, the challenges that employees face have become more diverse and intricate. In pursuit of organizational interests, employees may engage in behaviors that contravene societal values and legal standards. Umphress et al. (2010) were the first to define such conduct as unethical pro-organizational behavior (UPB). UPB embodies the contradictory characteristics of being both “pro-organizational” and “unethical” (Umphress et al., 2010). From organizational perspective, UPB may yield short-term benefits (Chen et al., 2023; Jiang and Xie, 2024; Khawaja et al., 2023; Umphress and Bingham, 2011; Wang et al., 2022; Xu et al., 2024; Zhang and Du, 2023). However, in the long term, these apparent advantages often transform into severe negative consequences (Baker et al., 2019; Umphress et al., 2010). A prominent example is the 2015 Volkswagen Group emissions scandal (Jung and Sharon, 2019), wherein senior engineers developed a “defeat device” to manipulate emissions test results, enabling vehicles to pass inspections under the guise of high environmental standards. Similarly, during the 2022 COVID-19 pandemic in China, Yuyue Medical-a leading manufacturer of medical devices-faced intense public backlash and regulatory scrutiny when its responsible executives drastically raised oximeters prices by over 500% to exploit soaring market demand, prioritizing corporate profits over public health needs. Although such actions may temporarily boost organizational performance, their inevitable exposure typically results in devastating outcomes, including irreparable reputational damage, substantial financial penalties, legal liabilities, and diminished stakeholder trust. The escalating frequency of corporate ethical scandals rooted in UPB has increasingly drawn the attention of scholars and organizational leaders to employee misconduct. While existing research on UPB has made significant strides in identifying its antecedents, there remains a considerable gap in understanding its long-term organizational consequences (Steele et al., 2023).

Existing studies have documented substantial heterogeneity in the impact of UPB on actors’ work attitudes, work behaviors, and work performance, with no consensus yet reached. These discrepancies primarily stem from the dual attributes of UPB—its organizational orientation and unethical nature—which activate distinct emotional mechanisms (e.g., pride, shame, guilt, psychological entitlement) and boundary conditions (e.g., ethical climate, moral identity). Specifically, one stream of research posits that UPB exerts a positive influence on actors’ work behaviors, particularly organization-oriented or other-oriented organizational citizenship behavior (OCB). Tang et al. (2020) argue that the pro-organizational essence of UPB may elicit feelings of pride among actors, enhance their organizational identification, and motivate them to take on additional responsibilities, such as maintaining the organization’s reputation. Additionally, actors may experience guilt for harming external stakeholders like customers, which in turn generates a compensatory drive to improve service delivery, such as addressing additional customer concerns. Conversely, another perspective suggests that UPB negatively affects work behaviors, leading to self-serving or counterproductive behaviors. For example, Chen et al. (2023) propose that UPB actors, by perceiving their actions as beneficial to the organization, may develop a heightened sense of psychological entitlement, justifying self-serving behaviors such as falsifying expense reports or reducing work effort. Similarly, Xu et al. (2024) argue that the unethical nature of UPB may induce feelings of shame, leading to emotional exhaustion and subsequent withdrawal behaviors, such as reduced commitment or task avoidance. Divergent views also exist regarding the impact of UPB on work attitudes. Gao (2019) posits that UPB can significantly enhance individuals’ job satisfaction, whereas Xia (2014) argues that UPB exerts a significant negative influence on individuals’ job satisfaction. Some researchers further contend that UPB may give rise to negative work attitudes, such as job burnout and turnover intentions (Xu et al., 2024). Yang et al. (2022) propose that the moral tension associated with UPB triggers cognitive dissonance, leading actors to experience conflicting emotions of pride and shame, which in turn undermines the stability of work attitudes - manifesting, for instance, as high engagement on 1 day and work avoidance the next. The discrepancies in how UPB affects work performance are primarily reflected in the time dimension. Yang et al. (2022) suggest that the sense of pride generated by UPB can drive actors to fully commit to their work and exert greater effort in the short term, thereby positively influencing short-term task performance. In contrast, Hao et al. (2023) assert that the feelings of shame or guilt induced by UPB may result in emotional exhaustion and sustained energy depletion over time, thereby negatively affecting long-term task performance. These conflicting findings hinder a clear understanding of the relationship between UPB and actors’ work attitudes, work behaviors, and work performance.

There remain certain aspects that require further clarification regarding the conditions, timing, and mechanisms through which UPB influences the actors’ work attitudes, behaviors and performance.

First, how does UPB affect actors’ work attitudes, behaviors, and performance? As previously discussed, theoretical analyses have indicated that UPB exerts an influence on these outcomes, and empirical studies have also obtained similar conclusions. However, the existing body of research is fragmented, necessitating a more comprehensive and systematic synthesis. Furthermore, the direction and intensity of the impact of UPB on actors’ work attitudes, behaviors, and performance remain ambiguous. For example, regarding the relationship between UPB and job satisfaction, Gao (2019) reports a positive association, whereas Xia (2014) finds a negative one. Similarly, regarding UPB and customer-oriented OCB, Tang et al. (2020) observe a positive correlation, while Gong (2024) identifies a negative one. Moreover, the correlation coefficients between UPB and the same type of outcome variables vary considerably. According to Abraham and Russell (2008), the strength of a correlation between variables can be categorized as weak (0.10–0.29), moderate (0.30–0.49), and strong (0.50 and above). For example, although UPB and OCB are generally positively correlated, the strength of this relationship ranges from weak to strong (Alqhaiwi, 2024; Tang et al., 2020; Zhang and Du, 2023).

Second, do these influences vary across cultures and genders? Are there differences attributable to statistical sampling methods? The boundary conditions of the impact of UPB on work attitudes, behaviors, and performance remain insufficiently understood. For example, there are significant differences between Eastern and Western cultural backgrounds. Western culture emphasizes individualism and personal autonomy, whereas Eastern culture prioritizes collectivism and emotional self-regulation. Consequently, the acceptance and perception of UPB may differ across these cultural backgrounds. Although existing meta-analyses evidence suggests that organizational tenure moderates the impacts of UPB on actors (Jiang and Xie, 2024), no empirical studies have yet investigated the moderating effects of sampling methods, cultural backgrounds, and gender. This gap limits the understanding of both scholars and practical managers regarding the impact of UPB.

Third, how does UPB influence actors’ work attitudes, behaviors, and performance? Although prior empirical studies have examined the mechanisms through which UPB affects individual work effectiveness, these mechanisms remain fragmented. Furthermore, while existing meta-analyses on UPB have laid important foundations for understanding this construct, they differ significantly in research focus and theoretical orientation, highlighting the need for further exploration (Chen, 2021; Helm, 2013; Hyun et al., 2021; Kashif et al., 2017; Peng et al., 2019; Wang et al., 2024; Zhang et al., 2023). Jiang and Xie (2024) adopted a dual-role perspective (actors vs. observers) to explore how actors’ UPB affects their own counterproductive work behaviors, OCB, and observers’ UPB, with a focus on cognitive and motivational mediators such as psychological entitlement, guilt, and moral disengagement. However, this analysis lacks a comprehensive investigation of how UPB affects actors’ work attitudes (e.g., job burnout, turnover intention) and work performance. They emphasize cognitive and motivational mediators while overlooking the emotional transmission mechanism of UPB, failing to systematically examine how UPB triggers emotional responses and further influences work outcomes.

To fill these gaps, this study adopts Affective Events Theory (AET) as the core theoretical framework and employs Meta-Analytic Structural Equation Modeling (MASEM) to systematically integrate 44 empirical studies, aiming to explore the relationship between UPB and actors’ work attitudes, work behaviors, and work performance. The objective is to uncover the underlying mechanism and boundary conditions that moderate the impact of UPB on these outcomes. This study aims to achieve the following advancements and innovations: (1) To systematically clarify the specific impact of UPB on actors’ work attitudes, behaviors, and performance by integrating the previous dispersed quantitative studies, thereby generating synthesized and reliable conclusions. (2) To determine the moderating factors that influence the relationship between UPB and actors’ work outcomes. By examining methodological, cultural, and gender-related variables, this study seeks to explain heterogeneity in existing findings and enrich the current understanding of UPB’s impact. (3) To explore the mediating role of emotional factors in the relationship between UPB and actors’ work outcomes, and to offer empirical support from multiple studies for mitigating UPB and minimizing its negative consequences for individuals or organizations.

## 2 Theoretical analysis and research hypotheses

### 2.1 Variable definition

The concept of UPB originated from the observation of phenomena in management practice. Scholars have identified that employees may engage in harmful behaviors in the workplace with the intention of benefiting their organizations. This has led to the emergence of a research perspective centered on pro-organizational motives within the study of unethical behavior in organizations (Pinto et al., 2008; Vardi and Wiener, 1996). Vardi and Wiener (1996) categorized misbehavior in organizations into three types based on differing behavioral intentions, one of which specifically aimed at benefiting the organization. This finding indicated that such misbehaviors are prevalent and may potentially harm the organization. In their study on organizational corruption, Pinto et al. (2008) regarded the beneficiaries of the behavior as a key dimension for classifying behavior types. They argued that corruption committed for the benefit of the organization constitutes a significant aspect of organizational corruption, and variations in this dimension may lead to differing attitudes toward corrupt behaviors within the same organization. Synthesizing the aforementioned research findings, Umphress and Bingham (2011) formally proposed the concept of UPB. They emphasized that understanding unethical behaviors with pro-organizational attributes from the perspective of “the motivation behind unethical behaviors” holds both theoretical value and practical significance. This concept comprehensively accounts for the external characteristics of UPB, encompassing both the “unethical” and “pro-organizational” traits. Therefore, this study also adopts this definition to delineate UPB. Most of the literature utilized for data analysis in this study employs the unidimensional measurement scale of UPB developed by Umphress et al. (2010), which is widely recognized as a standard instrument in this field. Specifically, this scale measures UPB through two approaches: the first evaluates respondents’ willingness to engage in UPB, and the second examines their actual UPB behavior.

This study investigates the impact of UPB on actors’ work attitudes, behaviors, and performance. The research design follows three core logics, as detailed below: First, guided by the principles of meta-analytic methodology, this study incorporates three categories of outcome variables—work attitudes, work behaviors, and work performance—to evaluate actors’ work effectiveness. Second, drawing on the approach outlined by Harrison et al. (2006), variables with similar conceptual meanings are aggregated into broader categories to enhance the predictive validity of the relationships being analyzed. Consistent with this approach, this meta-analysis adopts this modular classification approach, aggregating scattered outcome variables into unified categories. For example, workplace deviant behavior, silent behavior, and organizational citizenship behavior are classified under the overarching category of “work behaviors.” Finally, both work behavior and work attitude are further differentiated into multiple sub-dimensions to explore the nuanced influence of UPB across various aspects. Specifically, the three aforementioned categories of indicators encompass five specific sub-indicators: positive work attitudes, negative work attitudes, workplace deviant behavior, organizational citizenship behavior, and work performance.

Specifically, work attitude refers to an individual’s emotional or evaluative responses toward their work, shaped by cumulative learning experiences. It comprises subjective emotional, cognitive, and behavioral components related to work (Smith et al., 2013). The core characteristic of work attitude is “valence”—the positive or negative nature of evaluation (Ajzen and Fishbein, 2000). Accordingly, work attitudes can be broadly classified into positive work attitudes and negative work attitudes in this study. Positive work attitudes are typically expressed through recognition of work, job satisfaction, and willingness to commit, such as statements like “I enjoy my work” or “I am willing to put in extra effort for my work.” Negative work attitudes, on the other hand, manifest as job dissatisfaction, emotional exhaustion, and avoidance behaviors, such as statements like “I feel tired of my work” or “I want to leave this job.” Drawing from existing literature, constructs such as job satisfaction, work wellbeing, and work engagement are categorized under positive work attitudes, whereas constructs like job burnout and turnover intention are categorized under negative work attitudes.

Based on the classic dichotomy of work behaviors in organizational behavior research—which distinguishes behaviors according to their compliance with organizational norms and directional impact on the organization (Spector and Fox, 2005)—work behaviors are categorized into two dimensions: organizational citizenship behavior and workplace deviant behavior. Organizational citizenship behavior (OCB) refers to voluntary behaviors that are not explicitly recognized by the formal reward system but benefit the organization (Organ, 2018). Its core characteristic is proactive value for the organization, and it is considered a constructive behavior. Workplace deviant behavior, by contrast, refers to behaviors that violate organizational norms and threaten the interests of the organization or its members (Robinson and Bennett, 1995). Its core feature is the active or passive infliction of harm on the organization, and it is regarded as a destructive behavior. In this framework, variables from existing literature—including organization-oriented citizenship behavior (OCB-O), customer-oriented citizenship behavior (OCB-C), socially-oriented citizenship behavior (OCB-S), ethical voice, and helping behavior—are classified under organizational citizenship behavior. Similarly, counterproductive work behavior, self-serving cheating, work withdrawal, customer deception, moral disengagement, and unethical behavior are categorized under workplace deviant behavior.

Work performance refers to the combination of behaviors, capabilities, and outcomes that reflect the extent to which an individual, team, or organization fulfills responsibilities expected under specific contextual conditions. It serves as a direct indicator of goal attainment (Koopmans et al., 2011), and reflects the value of an employee’s contribution to the organization—making it one of the most critical metrics for evaluating subordinate performance. Drawing from existing literature, this study incorporates variables under the broader construct of work performance, including task performance, contextual performance, innovation performance, and work effort.

Furthermore, this study investigates the process mechanism of UPB’s impact on actors’ work attitudes, behaviors, and performance from the lens of AET. The proposed mediating variables include positive emotions and negative emotions. Positive emotions are defined as affective experiences characterized by pleasure and satisfaction, which tend to enhance individuals’ positive evaluations of themselves or their environment. Negative emotions, by contrast, are defined as affective experiences accompanied by distress and unease, which strengthen individuals’ negative evaluations of themselves or their environment (Watson et al., 1988). In the context of UPB, positive emotions refer to positive emotional states (e.g., pleasure, pride, and sense of accomplishment) experienced by individuals in the workplace that are directly triggered by UPB. These emotions focus on immediate affective responses to UPB’s “pro-organizational attribute” (e.g., pride in contributing to organizational goals). Therefore, variables such as pride, accomplishment, and belongingness are categorized under positive emotions. Negative emotions refer to negative emotional states (e.g., guilt, shame, and anxiety) triggered by UPB’s “unethical nature,” as well as subsequent emotional exhaustion and psychological discomfort resulting from moral cognitive conflicts. Variables such as shame, guilt, anxiety, and moral conflict are categorized under negative emotions.

### 2.2 Theoretical foundation

Current research on the consequences of UPB primarily relies on multiple theoretical frameworks, each with inherent limitations: Emotion Appraisal Theory and Social Cognitive Moral Theory focus on the indirect elicitation of emotions through cognitive appraisal, emphasizing individuals’ subjective interpretations of events (e.g., Xu et al., 2024) argue that the evaluation of the ethical attributes of UPB triggers shame or pride]. Self-Conscious Emotion Theory only examines the long-term associations between a limited set of emotions (pride, shame, guilt) and moral self-concept, making it difficult to explain short-term immediate emotional responses (e.g., Khawaja et al., 2023, exploration of UPB consequences among narcissistic employees). Moral identity theory focuses on the impact of static personality traits on UPB outcomes (Gao et al., 2023). Conservation of Resources Theory conceptualizes emotions as byproducts of resource loss, focusing on the unidirectional effects of specific negative emotions (Liu et al., 2021).

Affective Events Theory provides a robust theoretical foundation for explaining the interrelationships among workplace events, emotional experiences, work attitudes, and behaviors, making it the ideal framework for analyzing UPB’s consequences (Weiss and Cropanzano, 1996). Rooted in AET’s core propositions, salient workplace occurrences (termed affective events) act as the primary triggers of individuals’ immediate, discrete emotional reactions, and these emotions in turn shape their long-term work attitudes and subsequent behavioral outcomes. Depending on an affective event’s valence and inherent attributes, it can elicit divergent emotional responses—positive emotions such as pleasure and pride or negative emotions like anxiety and shame—and distinct emotion types further drive heterogeneous work outcomes. As a unique and salient workplace affective event, UPB carries dual attributes that trigger this kind of mixed emotional response. Its pro-organizational nature may foster positive emotions (e.g., satisfaction, pride) stemming from perceived contributions to collective interests, while its unethical essence can evoke negative emotions tied to moral conflict and norm violation. These dual emotional outcomes then give rise to the complex array of attitudinal and behavioral consequences associated with UPB. Empirical research has further validated AET’s utility in unpacking UPB-related psychological processes. For instance, Chen and Zhang (2024) leveraged AET to reveal that shame induced by UPB’s unethicality mediates the link between UPB and internal whistle-blowing behavior, and Zhang (2021) utilized the framework to identify mixed emotions as the key mediator of UPB’s impact on work wellbeing.

Beyond its explanatory power for these specific relationships, AET also addresses the limitations of alternative theoretical frameworks for UPB research. First, AET emphasizes that work events (including UPB) directly and immediately trigger emotions without relying on complex cognitive mediators. Tang et al. (2020) confirmed through Experience Sampling Method that employees experience immediate feelings of pride or guilt after engaging in UPB, which aligns closely with AET’s logic of “events directly triggering emotions.” Second, AET regards emotion as an independent core mediating variable, emphasizing its critical role between events and outcomes, rather than viewing emotions as a subsidiary of other psychological processes. This highlights the indispensable role of emotions in the transmission process and addresses the limitations of prior theories that underemphasized the mediating function of emotions. Third, existing theories predominantly focus on a limited range of emotions (e.g., pride, shame, and guilt), whereas AET accommodates a broader spectrum of emotional responses (e.g., anxiety and moral anger), thereby offering a more comprehensive explanation of the multiple impacts of UPB on work outcomes. For example, Jiang et al. (2023) demonstrated that UPB can reduce work effort through the mediating effect of anxiety. To sum up, through focusing on the dynamic, immediate, and complex association of “event—emotion—result,” AET makes up for the shortcomings of existing theories in terms of stativity, simplicity of emotions, and separation of short-term and long-term, providing a more realistic theoretical framework for analyzing the impact of UPB on work attitudes, behaviors, and performance. Accordingly, this study adopts AET as its theoretical foundation to elucidate the mechanism through which UPB influences actors’ work attitudes, behaviors, and performance.

### 2.3 UPB and work outcomes

#### 2.3.1 UPB and actors’ work attitudes

UPB exerts a multifaceted impact on actors’ work attitudes, presenting a complex pattern that is reflected in the extant literatures. From a potential positive perspective, UPB is intended to contribute to collective organizational interests and may yield significant short-term benefits to the organization. The altruistic values embedded in UPB may earn individual recognition and respect from the organization and its members, thereby fostering a sense of personal fulfillment and job satisfaction (Gao, 2019). Drawing on social exchange theory, employees who engage in UPB demonstrate a high level of loyalty to the organization and team. Under the principle of reciprocity, organizations are thus more likely to satisfy actors’ needs for autonomy and growth, further enhancing their job satisfaction (Wang et al., 2021). Additionally, Judge et al. (2023) proposed that when individuals develop intrinsic motivation and increased work interest when engaging in challenging and complex tasks. The paradoxical nature of UPB implies that it is a challenging endeavor, potentially boosting job satisfaction.

However, the core of positive work attitudes lies in individuals’ positive evaluation of and active engagement in work, whose formation depends on the alignment between personal values and the values of work or organization. The “unethical” essence of UPB undermines this value alignment, ultimately exerting a negative impact on positive work attitudes. Specifically, Xu et al. (2024) noted that in a low ethical climate, UPB may lead actors to perceive their contributions to the organization, potentially triggering short-term pride and thus temporarily enhancing work identification and engagement. However, such positive emotions are context-dependent and transient. Once the ethical climate is strengthened (e.g., in a high ethical climate) or individuals’ moral reflection intensifies, pride is replaced by shame or guilt, rendering the short-term improvement of positive attitudes unsustainable (Tang et al., 2020). Yang et al. (2022) further indicated that UPB triggers persistent moral cognitive conflict, wherein actors desire to identify with the organization while experiencing a sense of alienation due to violating their own moral standards. This conflict diminishes work meaningfulness (reducing job satisfaction) and depletes psychological resources (reducing work engagement), ultimately exerting a long-term inhibitory effect on positive work attitudes. Chen et al. (2023) emphasized that this phenomenon is particularly pronounced among individuals with high moral identity. Similarly, Liu et al. (2021) argued that the unethicality of UPB fundamentally conflicts with individuals’ deep-seated moral values (e.g., integrity, fairness). Although individuals may experience temporary satisfaction from actions perceived as beneficial to the organization, such behavior constitutes a form of self-moral betrayal in the long term, which significantly undermines positive job evaluations.

From the perspective of negative work attitudes (e.g., job burnout, turnover intention)—characterized by individuals’ negative evaluation of and avoidance tendency toward work—their formation is directly related to “emotional exhaustion” and “self-organization conflict.” UPB significantly strengthens negative work attitudes by triggering negative emotions and psychological resource depletion, in line with AET’s event-emotion-outcome chain. Specifically, the unethicality of UPB, on one hand, elicits actors’ self-denial and shame; prolonged feeling of shame can directly exacerbate emotional exhaustion, ultimately leading to job burnout (Xu et al., 2024; Du, 2019; Memon et al., 2017). On the other hand, actors may experience regret for their behavior, manifesting as guilt. Guilt intensifies the moral conflict between the self and the organization, causing cognitive dissonance and psychological discomfort (Yang et al., 2022), thereby increasing the likelihood of turnover intention (Liu et al., 2021; Mesdaghinia et al., 2019). Furthermore, empirical evidence indicates that after engaging in UPB, employees may assess the severity of potential punishment and the organization’s affective commitment. If they anticipate severe punishment from the organization or leaders and fail to obtain emotional support or recognition, they may develop disappointment toward the organization, further increasing turnover intention (Liu et al., 2024).

Accordingly, we propose the following Hypotheses:

*H1a*: UPB is negatively correlated with positive work attitudes.

*H1b*: UPB is positively correlated with negative work attitudes.

#### 2.3.2 UPB and actors’ work behaviors

Organizational Citizenship Behavior (OCB) refers to informal, voluntary positive behaviors not explicitly mandated by organizational norms but that contribute to organizational effectiveness (Organ, 2018). Its core drivers are “organizational identification” or “compensatory motivation”—two mechanisms that are directly activated by UPB’s dual attributes (pro-organizational orientation vs. unethical nature) through distinct emotional and social exchange pathways. Specifically, the pro-organizational nature of UPB elicits feelings of pride among actors, which strengthens their organizational identification and fosters a proactive willingness to engage in OCB. For example, through experience sampling, Tang et al. (2020) found that pride triggered by UPB is significantly positively correlated with OCB-O, manifested as proactively maintaining organizational reputation and helping colleagues solve problems. From the perspective of social exchange theory, Gong (2024) further demonstrated that UPB functions as a form of reciprocal behavior in manager-employee relationships: employees who engage in UPB are motivated to participate in OCB-O through enhanced manager-employee exchange quality.

The unethical nature of UPB induces psychological states such as moral deficiency, guilt, and shame among actors, prompting them to engage in voluntary compensatory behaviors to mitigate these negative feelings (Jiang et al., 2023; Tang et al., 2020; Yang et al., 2022; Liu et al., 2019). Examples of such compensatory OCB include internal whistle-blowing (Chen and Zhang, 2024), proposing constructive suggestions (Wang et al., 2022), helping behaviors, and innovative behaviors (Shin et al., 2024). Similarly, customer-oriented employees who recognize that their UPB has harmed customers are more likely to engage in OCB-C out of guilt—such as improving customer service and enhancing in-role service performance (Alqhaiwi, 2024; Chen et al., 2023; Gong, 2024). Given that UPB often benefits the organization at the expense of external stakeholders, actors may also engage in OCB-S by compensatory motivation, such as participating in charitable activities (Zhang and Du, 2023) or adopting pro-environmental behavior (Zhao and Qu, 2022).

Deviant Workplace Behavior (DWB) refers to voluntary behaviors by employees that violate organizational norms and threaten the interests of the organization or its members (Robinson and Bennett, 1995)—a prevalent negative behavior in organizational settings. DWB can be categorized into minor and major deviance (Griffin and Lopez, 2005), encompassing low-intensity, ambiguously intended workplace incivilities (e.g., discrimination, deception, prejudice and arrogance) and high-intensity, explicitly intended counterproductive work behaviors and antisocial behaviors (Baharom et al., 2017; Ahmad et al., 2020; Lubbadeh, 2021). The core drivers of DWB are “self-interest priority” or “moral disengagement,” both of which are reinforced by UPB’s dual attributes through emotional and cognitive mechanisms. First, the pro-organizational nature of UPB leads actors to perceive that they have “incurred extra costs for the organization,” triggering psychological entitlement—a belief that the organization owes them reciprocation. This sense of entitlement rationalizes self-serving behaviors, as actors frame deviance as a “deserved reward” for their pro-organizational contributions. For example, Chen et al. (2023) found that UPB significantly and positively predicts self-serving cheating through psychological entitlement, manifested in deviant behaviors such as falsifying expenses, concealing work errors and shirking job responsibilities. Second, the unethical nature of UPB weakens actors’ moral self-constraints. Repeated engagement in UPB may lead actors to internalize the cognition that ethical boundaries can be violated for organizational interests, thereby activating their moral disengagement mechanism (Fehr et al., 2019). This moral disengagement generalizes to other work domains, reducing the psychological cost of engaging in deviant behaviors and leading to more frequent DWB—such as slacking off, deceiving customers, or undermining colleagues. For instance, Yang et al. (2022) noted that moral cognitive dissonance triggered by UPB reduces actors’ guilt over deviant behaviors, indirectly promoting work withdrawal. Third, although UPB may induce guilt, when actors cannot alleviate this guilt through compensatory positive behaviors (e.g., the organization lacks formal moral repair channels or fails to recognize their compensatory efforts), they may resort to “reverse compensation” to release psychological pressure. This often manifests as retaliatory deviant behaviors directed toward the organization, such as deliberately reducing work quality, withholding critical information, or violating organizational procedures. Liu et al. (2021) confirmed this mechanism through a longitudinal study, finding that the incidence of DWB among UPB actors who failed to achieve moral repair in the long term was 23% higher than that of the control group in the long term.

Accordingly, we propose the following Hypotheses:

*H2a*: UPB is positively correlated with DWB.

*H2b*: UPB is positively correlated with OCB.

#### 2.3.3 UPB and actors’ work performance

Work performance refers to the set of behaviors exhibited by individuals under specific situational conditions and the corresponding outcomes achieved through these behaviors (Kraska et al., 1991). It reflects the value of employees’ work outputs to organization goals and is widely recognized as a core outcome variable in organizational behavior research. Empirical studies indicate that the impact of UPB on work performance is inherently dynamic and context-dependent, presenting contradictory short-term and long-term effects driven by UPB’s dual attributes (Guo et al., 2024; Kraemer and Gouthier, 2014; Mo and Shi, 2017; Zhan and Liu, 2022).

Drawing on the principle of reciprocal fairness in social exchange, UPB—despite its unethical nature—functions as a pro-organizational behavior that can generate immediate benefits for organizational operations and managerial effectiveness (Gong, 2024). This pro-organizational intent facilitates the development of positive exchange relationships between actors and their supervisors: organizations and leaders often reciprocate UPB with tangible and intangible “rewards,” such as increased compensation, enhanced trust, emotional support, and professional recognition. These reciprocal resources directly contribute to improving actors’ short-term work performance (Gao, 2019). Additionally, after engaging in UPB, employees may develop a heightened sense of psychological entitlement, framing their unethical contributions as integral to organizational success. This mindset motivates them to engage in more innovative performance behaviors (Shin et al., 2024).

In contrast, the unethical essence of UPB exerts a dominant, long-term negative impact on work performance through mechanisms of negative emotional arousal and psychological resource depletion. Hao et al. (2023) demonstrated that UPB indirectly reduces long-term task performance through the mediation of depressive emotions, with this effect being more pronounced among employees with high self-reflection. Highly self-reflective individuals are more likely to experience intense self-blame due to the moral conflict inherent in UPB, which further depletes cognitive resources and impairs work efficiency over time. Yang et al. (2022) noted that UPB’s dual nature triggers persistent moral cognitive dissonance. To alleviate such dissonance, employees become trapped in repeated struggles between “self-justification” and “moral reflection,” resulting in performance instability. This instability manifests as fluctuating productivity (e.g., high efficiency on some days and low efficiency on others), which ultimately lower overall performance evaluations in the long term.

Moreover, the exposure of UPB directly harms organizational reputation and indirect performance outcomes (Hao et al., 2023; Xu et al., 2024). For example, if customers discover deceptive practices or regulatory authorities investigate unethical conduct, the resulting damage to the organization’s brand image undermines market competitiveness and indirectly reduces individual performance opportunities (e.g., fewer sales leads, constrained resource access). Even in the absence of direct exposure, UPB’s accumulated “hidden harm”—such as eroded customer trust, damaged stakeholder relationships, or internal ethical climate deterioration—gradually emerges to constrain performance growth. For example, employees who deliberately conceal product defects to meet short-term sales targets may trigger a surge in customer complaints and reduced repurchase rates, ultimately leading to a significant decline in subsequent sales performance. Notably, while some studies suggest that UPB may temporarily enhance work effort (Jiang et al., 2023), this positive effect is highly context-dependent (e.g., low ethical climate, the short-term suppression of moral conflict) and unsustainable. Tang et al. (2020) confirmed this through longitudinal data, finding that the pride-mediated positive impact of UPB on performance persists for only 1–2 weeks, after which it is overshadowed by the shame-driven negative effect. Collectively, the short-term potential benefits of UPB are transient and context-bound, whereas its long-term negative effects are enduring, multifaceted, and dominant.

Accordingly, we propose the following Hypotheses:

*H3*: UPB is negatively correlated with work performance.

### 2.4 Moderating effects of potential variables

#### 2.4.1 Methodological factors

Different research designs and data collection methods often introduce variability in meta-analytic conclusions, as they capture distinct temporal dynamics and reduce or amplify biases (Howard et al., 2020). Consequently, methodological factors are widely recognized as critical moderators when examining bivariate relationships in meta-analyses. Longitudinal studies by Tang et al. (2020) and Hao et al. (2023) have consistently confirmed that the impact of UPB is inherently dynamic. Its “pro-organizational nature” may generate short-term positive effects, while long-term negative effects emerge as the cumulative harm of its “unethicality” becomes salient.

To further address common method bias (Podsakoff et al., 2012) and capture temporal nuances in UPB’s effects, this study investigates the moderating role of sampling time on the relationships between UPB and work attitudes, work behaviors, and work performance. Specifically, it examines whether the use of cross-sectional data (collected at a single time point) or multi-wave data (collected at multiple time points) in primary studies influences the magnitude and direction of the UPB-outcome relationships. Cross-sectional data typically captures immediate or short-term effects of UPB, leading to more positive effect sizes. In contrast, multi-wave data reflects long-term emotional accumulation and behavioral inertia, resulting in more negative effect sizes. For example, Tang et al. (2020)—a multi-wave study—found that the positive effect of UPB on performance diminishes over time and is eventually overshadowed by negative effects, whereas early cross-sectional studies on the UPB-OCB relationship may have overestimated positive impacts due to their failure to capture temporal dynamics. This supports the necessity of considering sampling time as a moderator factor. Based on this, the following hypothesis is proposed:

*H4a*: Sampling time moderates the relationships between UPB and work outcomes. Compared with multi-wave studies, cross-sectional studies show a weaker positive relationship between UPB and negative work attitudes, but a stronger positive relationship between UPB and OCB.

#### 2.4.2 Cultural background

Numerous meta-analyses have demonstrated that cultural background moderates the correlations between organizational constructs. National culture represents a collective programming of the mind that distinguishes members of one group from those of others, shaping individuals’ cognition, emotions, and behaviors in distinct ways. Notably, Eastern and Western cultures exhibit fundamental differences in core dimension (e.g., collectivism vs. individualism, high vs. low power distance) that significantly influence organizational management and moral judgment (Hofstede et al., 2010). In Eastern cultural contexts (characterized by collectivism and high-power distance), the pro-organizational attribute of UPB is more likely to be perceived as legitimate and socially desirable, while the moral conflict associated with its unethical nature is weakened. This cultural orientation strengthens the positive effect of UPB on OCB and weakens the negative inhibition of UPB on DWB (Chen et al., 2023; Xu et al., 2024). In contrast, Western cultural contexts (characterized by individualism and low power distance) emphasize individual moral autonomy and personal responsibility: the unethical essence of UPB is more likely to trigger intense guilt and shame, resulting in a stronger positive relationship between UPB and negative work attitudes, and a more significant negative relationship between UPB and work performance (Tang et al., 2020). Additionally, Xu et al. (2024), using a Chinese sample (Eastern context), found that the moderating effect of ethical climate on UPB outcomes was weaker than that reported in Western studies. This confirms that culture shapes moral judgment and subsequent behavioral responses to UPB. Therefore, the impact of UPB on actors’ work attitudes, work behaviors, and work performance is inherently intertwined with cultural background. Accordingly, Hypothesis H4b is proposed:

*H4b*: Cultural background moderates the relationships between UPB and work outcomes. Compared with Western cultural contexts, Eastern cultural contexts exhibit stronger relationships between UPB and negative work attitudes, OCB, and DWB.

#### 2.4.3 Gender

As a core individual-level difference variable, gender’s moderating role in ethical behavior and its subsequent outcomes can be explained through the core chain of AET. The fundamental logic is as follows: gender differences shape the sensitivity, experiential intensity, and expression patterns of moral emotions (Eagly and Sczesny, 2019), thereby influencing the pathway through which UPB affects work outcomes.

Gender socialization and societal role expectations lead to systematic differences between men and women in the emotional encoding of and responses to moral events, and these differences are particularly pronounced in the context of UPB. The female gender role is defined by “warmth and relationship orientation”; this role expectation inclines women to integrate moral goodness into their self-identity. When women engage in UPB—behavior that carries the dual attributes of “pro-organizational” and “unethical”—they are more likely to trigger self-conscious moral emotions (e.g., guilt, shame). In contrast, the male gender role emphasizes “agency and competitiveness,” leading men to be more inclined to suppress negative emotions induced by unethical behavior. Men thus exhibit weaker awareness of moral conflict regarding UPB and may even rationalize such behavior (Kundro and Rothbard, 2023; Ward and King, 2018). Specifically, after engaging in UPB, women’s sense of guilt drives cognitive dissonance (i.e., “inconsistency between behavior and self-moral identity”), while shame elicits withdrawal-related psychological responses. Men, however, are more likely to experience pride due to the “pro-organizational” outcomes of UPB and simultaneously suppress potential negative emotions, thereby reducing the psychological strain caused by moral conflict. This gender-based difference forms the core logic of gender’s moderating effect, which aligns with the core tenet of AET—that “individual differences shape emotional responses.”

Empirically, meta-analytic evidence from Dadaboyev et al. (2024) demonstrates that gender exerts a significant moderating effect on the relationship between organizational identification and UPB: the positive association between these two variables is significantly stronger in male samples than in female samples. This finding further confirms that men exhibit lower moral sensitivity to UPB and a stronger tendency to rationalize it in their emotional processing. Additionally, a cross-cultural study by Leonidou et al. (2013) found that even across different cultural contexts, women impose harsher moral condemnation on UPB than men. This attitudinal difference essentially stems from gender-based variation in the intensity of emotional experiences: women have a lower threshold for negative emotional responses to moral violations, whereas men exhibit more prominent positive emotional encoding of pro-organizational behavior.

Based on the above theoretical logic, gender indirectly influences the strength of the associations between UPB and work attitudes, behaviors, and performance by moderating the type and intensity of emotional responses triggered by UPB. A meta-analysis by Nohe et al. (2022) confirms that women express harsher moral condemnation of unethical behavior, and this attitude further amplifies the negative impact of UPB on their work attitudes. In contrast, men’s emotional suppression mechanisms buffer this negative association. Two empirical studies conducted in Poland by Gigol (2021) also revealed that after engaging in UPB, women are more inclined to repair their moral self-image through positive work behaviors, whereas men show no significant subsequent behavioral adjustments. Khawaja et al. (2023) reported that following the commission of unethical pro-organizational behavior, women’s shame scores were 12.3% higher than men’s, and women were more likely to engage in subsequent remedial behaviors (e.g., the interpersonal dimension of Organizational Citizenship Behavior, OCB-I). Therefore, the following hypothesis is proposed:

*H4c*: Gender moderates the relationships between UPB and work outcomes. Compared with men, women exhibit a stronger moderating effect on the relationships between UPB and negative work attitudes, as well as between UPB and OCB, but a weaker moderating effect on the relationship between UPB and DWB.

### 2.5 Mediating role of emotions

AET posits that both positive and negative workplace events elicit distinct emotional and cognitive responses in individuals. Specifically, negative events hinder goal attainment and induce negative emotions, while positive events facilitate goal attainment and evoking positive emotions (Dimotakis et al., 2011). The dual nature of UPB enable actors to experience concurrent positive and negative emotions. These positive and negative emotions have been theoretically and empirically validated as key mediators linking UPB to various outcome (Tang et al., 2020; Zhang and Du, 2023; Liao et al., 2024). Notably, while the present meta-analysis reveals no-significant relationships between UPB and positive work attitudes or work performance, the mediating role of emotions in other UPB-outcomes linkages remains robustly supported.

The “pro-organizational” nature of UPB positions it as a positive affective event, primarily triggering positive emotions such as pride. Actors often perceive UPB as a reflection of their alignment with organizational goals, signaling their commitment as “good employees” who safeguard organizational interests (Oveis et al., 2010; Xu et al., 2024). Positive emotions are generally associated with favorable work outcomes (Lan et al., 2022). When individuals attribute success or positive events to their own abilities or efforts, the resulting positive emotion shapes subsequent work behaviors (Seo et al., 2004). Employees’ desire to maintain pride in their work and organization can enhance job satisfaction, further motivating increased work engagement and driving high-quality work performance (Frone, 2015; Xu et al., 2024).

Beyond performance-related outcomes, positive emotions induced by UPB may also promote OCB. To sustain the temporary self-enhancement derived from pride, individuals may engage in additional prosocial actions targeting the organization or customers (Tang et al., 2020). Furthermore, positive emotions serve a protective function by inhibiting negative work outcomes: they alleviate job burnout, reduce turnover intention (Zhang et al., 2022), and mitigate counterproductive work behaviors (Spector and Fox, 2002; Sulea et al., 2012; Chiu and Tsai, 2006).

Conversely, the “unethical” core of UPB classifies it as a negative affective event, triggering negative emotions such as shame (denial of one’s moral integrity) and guilt (regret over behavioral consequences). From the perspective of AET, these negative emotions will further shape employees’ work attitudes and behaviors by affecting their internal psychological resource states. Existing research has shown that negative emotions triggered by workplace events can be regarded as a form of psychological resource consumption (Hobfoll et al., 2018), it consistent with the resource-related perspective of the Conservation of Resources Theory. When employees experience negative affect, they must allocate limited psychological resources to alleviate discomfort, leading to emotional exhaustion (Barling and Macintyre, 1993) and subsequent negative work attitudes such as job burnout and turnover intention (Vandevala et al., 2017; Cohen and Abedallah, 2015). Emotional exhaustion may also reduce self-behavioral capacity, increasing the likelihood of DWB such as retaliation or fraud against organizational members (Chen and Chen, 2023; Zhang and Du, 2023). Notably, negative emotions (e.g., shame or guilt) induced by UPB can also motivate proactive compensatory behaviors. To achieve inner purification and rebuild their moral image, individuals may engage in charitable activities (Zhang and Du, 2023), pro-environmental behaviors (Zhao et al., 2024), OCB-O (Jiang et al., 2023; Shin et al., 2024), and OCB-C (Alqhaiwi, 2024; Gong, 2024).

In summary, this study proposes Hypothesis 5:

*H5a*: UPB indirectly reduces negative work attitudes and indirectly promotes OCB and DWB via positive emotions.

*H5b*: UPB indirectly promotes negative work attitudes and OCB, and indirectly reduces DWB via negative emotions.

## 3 Meta-analysis procedure

### 3.1 Literature search and screening

To ensure the rigor and comprehensiveness of the meta-analysis, this study followed the systematic guidelines proposed by Cooper (2017) for retrieval and screening, consisting of three core steps: (1) defining relevant search terms to capture all potential studies; (2) applying predefined inclusion criteria to screen retrieved literature; (3) selecting eligible studies for subsequent data analysis.

A systematic search strategy was adopted using a combination of keywords related to the core construct (UPB), mediating variables (emotions), and outcome variables. The search terms include “unethical pro-organizational behavior/UPB/positive emotion/positive affection/pride/proud/negative emotion/negative affection/shame/guilt/regret/workplace wellbeing/job satisfaction/work engagement/job burnout/turnover intention/deviant workplace behavior/counterproductive behavior/fraud behavior/unethical behavior/organizational citizenship behavior/helping behavior/ethical voice/pro-environment behavior/customer service behavior/work performance/task performance/innovative behavior/leader-member exchange.” A comprehensive search was conducted across multiple academic databases, including Scopus, Web of Science, Taylor and Francis, PsycINFO (EBSCO), Google Scholar, Elsevier Science Direct, SAGE, Wiley, and Emerald. The retrieved literature covered journal articles, dissertations, conference proceedings, and book chapters. In parallel, corresponding Chinese keywords were used to conduct a comprehensive search of relevant Chinese literature in databases such as CNKI, Wanfang Data, and VIP Chinese Science and Technology Periodical Database. The final search was updated on July 30, 2025, yielding 5,584 preliminary studies including 952 from Chinese databases.

In the subsequent process of literature screening, two rounds of screening were conducted. Firstly, an initial screening was conducted by removing duplicate publications, and then reviewing the titles and abstracts of the remaining studies to retain those that might meet the meta-analysis inclusion criteria. Secondly, a full-text screening was carried out based on the following selection criteria: (1) Only empirical studies (quantitative designs) were included, excluding theoretical studies, review articles, and qualitative research. (2) All variables were measured using validated scales (e.g., Positive and Negative Affect Schedule for positive/negative emotions) with clearly reported dimension-specific or composite scores; (3) The study must report sample size and Pearson correlation coefficient (r). (4) The study must report at least one correlation coefficient illustrating the relationship between UPB and work attitudes, work behaviors, or work performance. (5) Duplicate use of the same survey data was not allowed. if a study was published both as a dissertation and in a peer-reviewed journal, only the journal article be included. (6) The study must focus on the individual-level consequences of UPB, excluding team-level or organizational-level UPB research. (7) Gray literatures (e.g., dissertations, conference proceedings) meet the above criteria was included to reduce publication bias. After full-text screening, 32 valid articles (including 6 Chinese-language studies) were retained for direct effect analysis.

**Figure 1:**
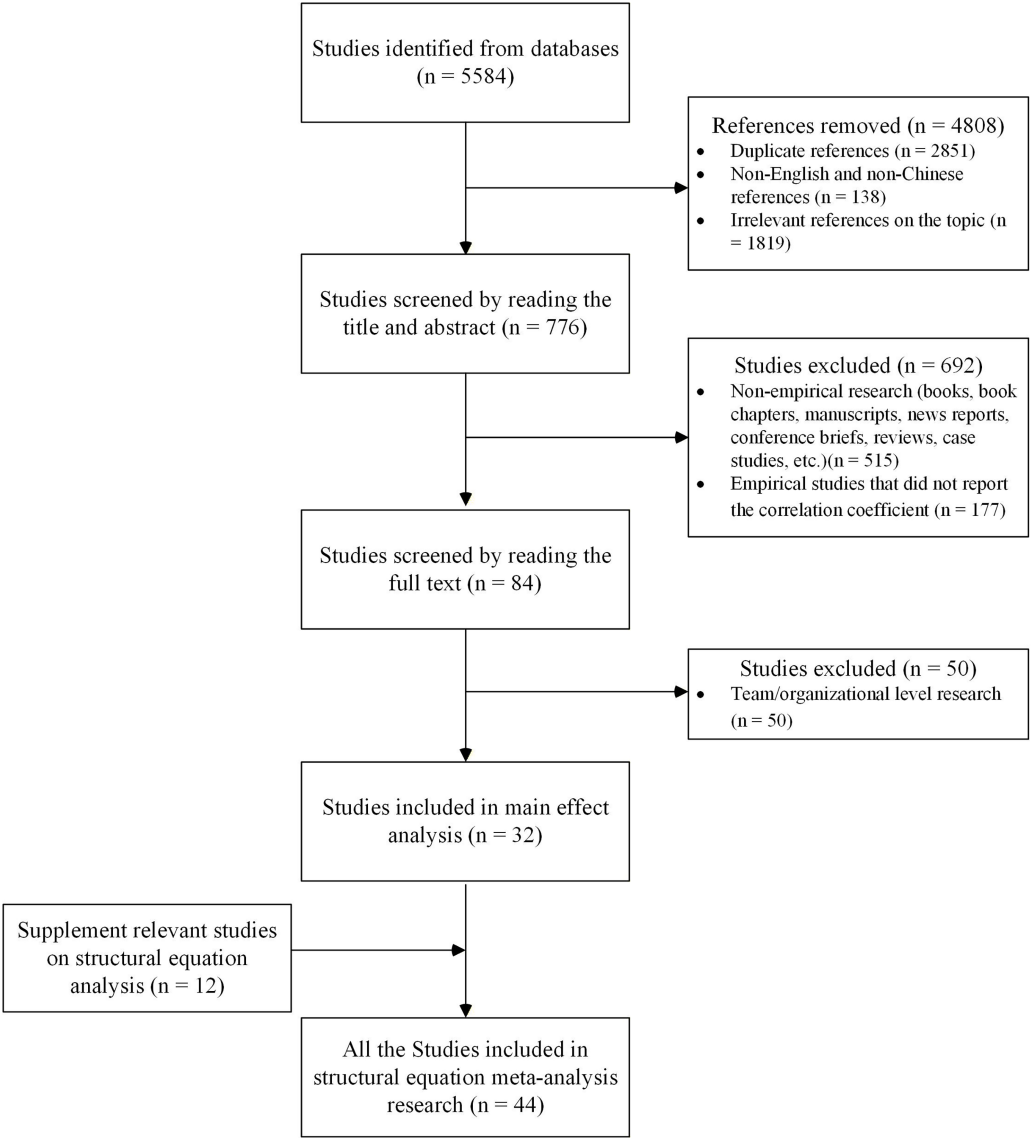
Article search and selection process.

To meet the data requirements for MASEM, supplementary literature retrieval was conducted following the approach of Yang and Li (2021). Specifically, first, if the previously collected literature reported pairwise correlation coefficients between mediating variables and work outcome variables, additional coding was performed for these coefficients. Second, joint searches were conducted using all relevant keywords related to mediating variables and work outcomes variables. Furthermore, backward retrieval was performed by screening the reference lists of the already included studies, as well as previous meta-analyses and review studies, to avoid missing relevant literature. This supplementary process yielded 12 additional valid articles, and ultimately 44 valid articles (including 4 gray literature sources) were included in the MASEM analysis, with the detailed literature search and screening process illustrated in Figure 1.

### 3.2 Data coding

First, two researchers collaboratively developed a coding protocol and manual, followed by independent coding of all eligible studies. Both descriptive information and effect size statistics were extracted during the coding process. Descriptive items included author(s), publication year, sampling period, cultural background, and the proportion of female participants; effect size statistics included sample size, Pearson correlation coefficient (r), and Cronbach’s alpha reliability coefficient for all focal scales. Second, when a study reported dimension-level correlation coefficients between the focal variables (e.g., UPB and outcome variables), these coefficients were aggregated into a single construct-level correlation using the formula proposed by Schmidt and Hunter (2015). Third, to maximize statistical power and ensure comprehensive synthesis, the initial related variables were broadly categorized into the following outcomes: UPB, negative emotions, positive emotions, negative work attitudes, positive work attitudes, DWB, OCB, and work performance. Fourth, measurement error correction was applied to all coefficients. Specifically, Formula (1) was used to adjust each reported correction coefficient ($r_{\mathrm{xy}}$) for unreliability in both the predictor and outcome measures, where $\alpha_{x}$ and $\alpha_{y}$ represent the Cronbach’s alpha coefficients of the independent variable and dependent variable scales, respectively. The corrected correlation coefficient ${\bar{r}}_{\mathrm{xy}}$ was derived as follows:


$${\bar{r}}_{\mathrm{xy}}=r_{\mathrm{xy}}/\sqrt{\alpha_{x}\alpha_{y}}$$

Fifth, for variables with unreported reliability coefficients, the weighted average reliability of the same construct from other included studies was used as a substitute (De Wit et al., 2012). Sixth, the samples included in the meta-analysis were drawn from nine countries/regions. We drew on the cultural value survey data from Hofstede et al. (2010), which rates each country or region on an individualism (IDV) scale ranging from 0 to 100. A higher score indicates stronger individualism and weaker collectivism, while a lower score reflects stronger collectivism and weaker individualism. In this study, we defined samples from regions with an IDV score <50 as Eastern cultural samples (high collectivism), covering countries/regions such as China, Pakistan, South Korea, Jordan, Côte d’Ivoire and Malaysia. Samples from regions with an IDV score >50 were labeled as Western cultural samples (low collectivism), with representative regions including the United States, Germany and Israel. The final inter-coder reliability reached 96.11%, and any coding discrepancies were resolved through discussion and consensus between the two coders. Ultimately, a total of 55 independent samples, 182 effect sizes, and a combined sample size of 18,074 were extracted for the final analysis.

### 3.3 Meta-analysis implementation

Following the methodological framework proposed by Cheung (2015), this study employed the MASEM method to simultaneously examine the direct effects of UPB on work outcomes and the mediating roles of positive emotions and negative emotions in these relationships. The MASEM implementation consisted of two sequential stages. In the first stage, CMA 3.0 software was used to conduct independent meta-analyses on the extracted empirical data. Specifically, publication bias was evaluated using fail-safe N, Egger’s test and Begg’s test. Heterogeneity across studies was assessed via Q-statistics and I^2^ values. Additionally, moderation analyses were performed to examine the potential moderating effects of methodological factors (e.g., sampling time), cultural background (Eastern vs. Western), and gender composition (proportion of female participants) on the relationships between UPB and work outcomes. The main effect analysis reports the number of effect sizes (k), total sample size (N), effect size (Pearson’s r), and 95% confidence interval (95% CI), ultimately generating a comprehensive correlation matrix of all focal variables. In the second stage, the correlation matrix derived from the first stage was imported into AMOS software to construct a structural equation model, which was then used to test the proposed mediating effects of positive and negative emotions.

## 4 Research results

### 4.1 Publication bias

**Figure 2:**
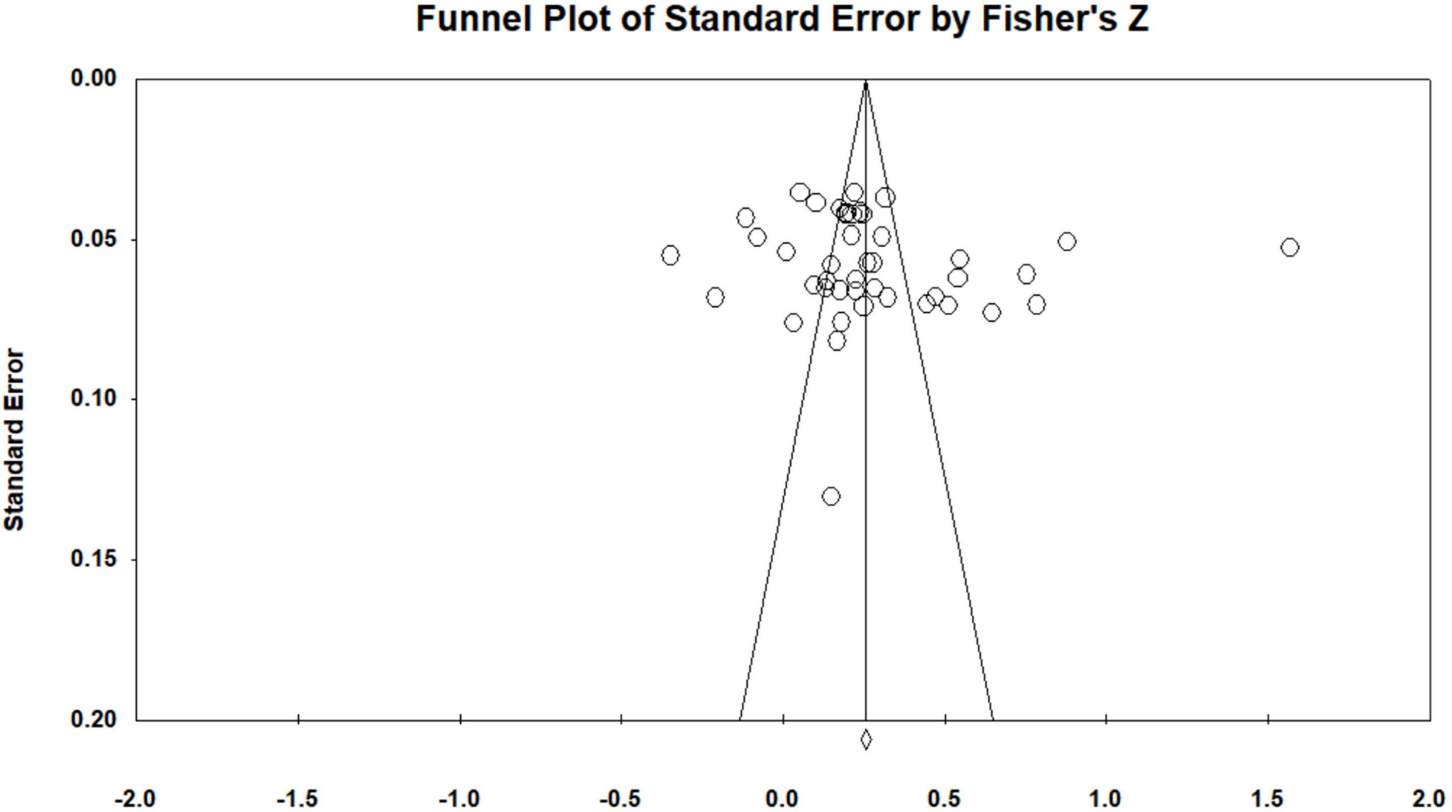
Funnel plot of standard error by Fisher’s *Z*.

A maxed qualitative-quantitative approach was employed to assess publication bias in the current meta-analysis. Initially, a funnel plot was used for visual inspection of publication bias in the relationship between UPB and overall work outcomes. As shown in Figure 2, the effect sizes of the UPB-outcome relationships across included studies were concentrated in the upper region of the funnel plot and distributed approximately symmetrically around the mean effect size, suggesting no severe publication bias initially. Subsequently, three quantitative tests—fail-safe N, Egger’s test and Begg’s test—were conducted to further verify publication bias. The fail-safe N was used to estimate the minimum number of unpublished studies required to negate the observed effects, with a threshold of fail-safe N > 5 k + 10 (where k denotes the number of effect sizes) indicating no significant publication bias. For Egger’s test and Begg’s test, a non-significant *p*-value (*p* > 0.05) signifies the absence of publication bias. As shown in Table 1, the fail-safe N values for all UPB-outcome relationships exceeded the critical value of 5 k + 10. Additionally, the Egger’s test and Begg’s test results yielded non-significant regression coefficients for all relationships (all *p* > 0.05). Collectively, the qualitative and quantitative evidences confirm that the conclusions of this study are robust and not affected by severe publication bias.

**Table 1:** Results of publication bias, heterogeneity test, and main effect analysis.

Correlation	Model	*k*	Effect size and 95% CI			Heterogeneity				Fail-safe N	5 *K* + 10	Begg’s	Egger’s
			r̄	Lower	Upper	Q	df (Q)	*p*	*I* ^2^			*p*	*p*
UPB-PE	R	7	0.273	0.159	0.381	77.696	6.000	0.000	92.278	445	45	0.293	0.521
UPB-NE	R	22	0.360	0.277	0.438	396.307	21.000	0.000	94.701	6,198	120	0.114	0.203
UPB-PWA	R	5	−0.078	−0.388	0.248	145.838	4.000	0.000	97.257	55	35	0.624	0.646
UPB-NWA	F	3	0.242	0.098	0.376	6.428	2.000	0.040	68.887	36	25	0.602	0.975
UPB-OCB	R	23	0.226	0.070	0.372	1153.389	22.000	0.000	98.093	2,523	125	0.673	0.792
UPB-DWB	R	15	0.232	0.127	0.331	213.532	14.000	0.000	93.444	829	85	0.216	0.283
UPB-WP	R	17	0.125	−0.049	0.292	688.934	16.000	0.000	97.678	489	95	0.174	0.307

R, Random effects; F, Fixed effects; k, number of correlations; r, corrected mean effect size; 95% CI, 95% confidence interval; Q, weighted sum of squared differences between individual study effects and the attenuated integrated effect size; I2, true variance/total dispersion. UPB unethical pro-organizational behavior, PE positive emotions, NE negative emotions, PWA positive work attitude, NWA negative work attitude, OCB organizational citizenship behavior, DWB deviant workplace behavior, WP work performance.

### 4.2 Heterogeneity test and main effect analysis

Heterogeneity, defined as variability in effect sizes across studies, may stem from differences in empirical design, research participants, sample characteristics (e.g., sample size), and outcomes measurements among the included studies. The results of heterogeneity testing determine the appropriate analytical model (fixed-effects vs. random-effects model) for subsequent meta-analyses. This study assessed heterogeneity using Q-statistics and I^2^ values. Specifically, a random-effects model was adopted if the Q-statistic was significant (*p* < 0.05), Q > k − 1 (where k denotes the number of effect sizes), and *I*^2^ > 0.75 (indicating high heterogeneity); otherwise, a fixed-effects model was used. As shown in Table 1, all UPB-outcome relationship exhibited significant heterogeneity (all *p* < 0.05, Q > k − 1, *I*^2^ > 75%), thus random-effects models were employed for all analysis.

As presented in Table 1, the main effect analysis results are summarized as follows: The effect size between UPB and positive work attitudes (PWA) was −0.078, with a 95% confidence interval (95% CI) that included zero, indicating a non-significant relationship. Thus, Hypothesis H1a was not supported. In contrast, UPB was significantly positively correlated with negative work attitudes (NWA) (r̄ = 0.242, 95% CI excluding zero), supporting Hypothesis H1b. For work behavior outcomes, UPB was significantly positively associated with both OCB (r̄ = 0.226, 95% CI excluding zero) and DWB (r̄ = 0.232, 95% CI excluding zero), thus supporting Hypotheses H2a and H2b. Regarding work performance, the correlation between UPB and work performance was non-significant (r̄ = 0.125, 95% CI including zero), leading to the rejection of Hypothesis H3. Given that the main effect tests confirmed no significant relationship between UPB and PWA or work performance, subsequent moderation effect tests and mediation analyses focused exclusively on the three significant outcome variables: NWA, OCB, and DWB.

To mitigate publication bias, four dissertations meeting the inclusion criteria were systematically incorporated into the analysis. A sensitivity analysis was conducted by excluding these dissertations, and the core effect sizes remained consistent (e.g., the correlation of between UPB and OCB was 0.226 in the full sample and was 0.219 after exclusion), confirming the robustness of the main effect results. Although dissertations may be associated with smaller sample sizes or less rigorous methodological designs, the application of strict inclusion criteria and sensitivity analysis has effectively mitigated potential risks of bias (Table 1).

### 4.3 Moderation effect test

Given the high heterogeneity observed in the main sizes of UPB-outcome relationships, moderation effect tests were conducted to identify potential sources of heterogeneity, focusing on three proposed moderators: sampling time, cultural background, and gender.

Since sampling time is a categorical variable, subgroup analysis was employed to examine its moderating role. As shown in Table 2, samples were divided into two subgroups: cross-sectional data and multi-wave data. The results showed that UPB was significant positively correlated with NWA in both subgroups: cross-sectional data (r̄ = 0.172, 95% CI = [0.044, 0.294]) and multi-wave data (r̄ = 0.279, 95% CI = [0.062, 0.470]), with neither 95% CI including zero. The strength of this relationship was more pronounced in the multi-wave subgroup compared to the cross-sectional subgroup. For the UPB-OCB relationship, the correlation was non-significant in the cross-sectional subgroup (r̄ = 0.211, 95% CI = [−0.091, 0.240], 95% CI includes zero) but significant in the multi-wave subgroup (r̄ = 0.269, 95% CI = [0.240, 0. 298], 95% CI excludes zero), indicating that sampling time moderates the UPB-OCB relationship. Regarding the UPB-DWB relationship, significant positive correlations were observed in both subgroups: cross-sectional data (r̄ = 0.209, 95% CI = [0.108, 0.305]) and multi-wave data (r̄ = 0.243, 95% CI = [0.062, 0.409]), with the cross-sectional subgroup showing a slightly more pronounced effect. Collectively, these results provide partial support for Hypothesis H4a.

**Table 2:** Moderating effect of sampling time on UPB and outcome variables.

Correlation	Heterogeneity			Subgroup	*k*	95% CI			Two-tailed test	
	Q	df	*p*			r̄	Lower	Upper	Z	*p*
UPB-NWA	0.000	0	1.000	Multi-wave	1	0.172	0.044	0.294	2.635	0.008
	4.381	1	0.036	Cross-sectional	2	0.279	0.062	0.470	2.498	0.012
UPB-OCB	1105.962	14	0.000	Multi-wave	15	0.269	0.240	0.298	17.087	0.000
	39.716	7	0.000	Cross-sectional	8	0.211	0.182	0.240	13.813	0.000
UPB-DWB	177.382	8	0.000	Multi-wave	9	0.243	0.062	0.409	2.612	0.009
	33.924	5	0.000	Cross-sectional	6	0.209	0.108	0.305	4.004	0.000

Cultural background (another categorical variable) was also examined via subgroup analysis, with samples divided into Eastern and Western culture subgroups. As shown in Table 3, the results revealed that UPB was significant positively correlated with NWA in both Eastern (r̄ = 0.276, 95% CI = [0.064, 0.464]) and Western (r̄ = 0.167, 95% CI = [0.009, 0.317]) culture subgroups, with neither 95% CI including zero. For the UPB-OCB relationship, significant positive correlations were observed in both Eastern (r̄ = 0.240, 95% CI = [0.060, 0.405]) and Western (r̄ = 0.153, 95% CI = [0.005, 0.295]) subgroups, with the effect size more pronounced in the Eastern culture subgroup. Similarly, UPB was significant positively correlated with DWB in both Eastern (r̄ = 0.199, 95% CI = [0.170, 0.229]) and Western (r̄ = 0.103, 95% CI = [0.044, 0.161]) subgroups, with a stronger effect in the Eastern culture subgroup. Overall, these results provide partial support for Hypothesis H4b.

**Table 3:** Moderating effect of cultural background on the relationships between UPB and outcome variables.

Correlation	Heterogeneity			Subgroup	*k*	95% CI			Two-tailed test	
	Q	df	*p*			r̄	Lower	Upper	Z	*p*
UPB-NWA	5.190	1	0.023	Eastern culture	2	0.276	0.064	0.464	2.532	0.011
	0.000	0	1.000	Western culture	1	0.167	0.009	0.317	2.065	0.039
UPB-OCB	1134.511	18	0.000	Eastern culture	19	0.240	0.060	0.405	2.603	0.009
	12.121	3	0.007	Western culture	4	0.153	0.005	0.295	2.022	0.043
UPB-DWB	129.994	10	0.000	Eastern culture	11	0.199	0.170	0.229	12.995	0.000
	75.037	3	0.000	Western culture	4	0.103	0.044	0.161	3.406	0.001

Since the proportion of females (a proxy for gender) is a continuous variable, meta-regression analysis was used to test its moderating effect. As shown in Table 4, gender significantly moderated both the UPB-DWB relationship (*β* = 0.001 < 0.05) and the UPB-OCB relationship (*β* = 0.032 < 0.05). Specifically, a higher proportion of females was associated with a weaker positive relationship between UPB and DWB, as well as between UPB and OCB. Due to insufficient data from included studies, the moderating effect of gender composition on the UPB-NWA relationship could not be verified. Therefore, these results provide support for Hypothesis H4c.

**Table 4:** Results of meta-regression analysis.

Moderator variable	Correlation	Beta	SE	95% CI		*P*
Gender	UPB-NWA	–	–	–	–	–
	UPB-OCB	0.011	0.005	0.001	0.021	0.032
	UPB-DWB	0.006	0.003	−0.001	0.012	0.001

### 4.4 Mediation effect test

Exploring mediating mechanisms based on confirmed main effects align with fundamental research logic. Drawing upon the results of the main effect analysis, existing evidence consistently demonstrates that, across diverse samples, UPB exerts a significant influence on NWA, OCB and DWB. Accordingly, this study focused on testing the mediating paths through which UPB influences these three outcome variables, with positive emotions and negative emotions as the focal mediators. The analysis followed the two-stage analytical procedure of MASEM proposed by Cheung (2015).

First, the correlation matrix for all focal variables is presented in Table 5, with the lower triangle reporting bivariate correlation coefficients (r̄), number of studies, sample size, and 95% confidence intervals (CIs) for each correlation. Specifically, UPB was correlated with positive emotion (r̄ = 0.273), negative emotion (r̄ = 0.360), NWA (r̄ = 0.242), OCB (r̄ = 0.226), and DWB (r̄ = 0.232); positive emotion was correlated with negative emotion (r̄ = − 0.080), NWA (r̄ = − 0.279), OCB (r̄ = 0.114), and DWB (r̄ = 0.138); negative emotion was correlated with NWA (r̄ = 0.354), OCB (r̄ = 0.110), and DWB (r̄ = 0.053). NWA were correlated with OCB (r̄ = − 0.2382) and DWB (r̄ = − 0.413). OCB was correlated with DWB (r̄ = − 0.115). For MASEM model estimation, the harmonic mean of the sample sizes corresponding to each effect size in the correlation matrix (*N* = 3,551) was used as the effective sample size, consistent with MASEM methodological standards.

**Table 5:** Meta-analytic correlation matrix.

Variable	UPB	PE	NE	NWA	OCB
UPB	–				
PE r̄ (*k, N*) 95% CI	0.273*** (*7, 3,628*) [0.159, 0.381]	–			
NE r̄ (*k, N*) 95% CI	0.360*** (*22, 8,773*) [0.277, 0.438]	−0.080* (*9, 4,130*) [−0.214, 0.058]	–		
NWA r̄ (*k, N*) 95% CI	0.242*** (*3, 577*) [0.098, 0.376]	−0.279*** (*6, 1,635*) [−0.586, 0.099]	0.354*** (*5, 1,170*) [−0.109, 0.690]	–	
OCB r̄ (*k, N*) 95% CI	0.226*** (*23, 8,065*) [0.070, 0.372]	0.114*** (*6, 3,437*) [0.014, 0.211]	0.110*** (*15, 5,746*) [−0.010, 0.227]	−0.382*** (*5, 1,339*) [−0.662, −0.009]	–
DWB r̄ (*k, N*) 95% CI	0.232*** (*15, 5,269*) [0.127, 0.331]	0.138*** (*5, 2,351*) [−0.067, 0.322]	0.053*** (*7, 3,191*) [−0.070, 0.175]	0.413*** (*3, 784*) [0.125, 0.637]	−0.115*** (*9, 3,165*) [−0.213, −0.015]

*k*, number of studies; *N*, combined sample size; r, mean effect size. *p < 0.05. **p < 0.01, ***p < 0.001; 95% CI, 95% confidence interval.

**Figure 3:**
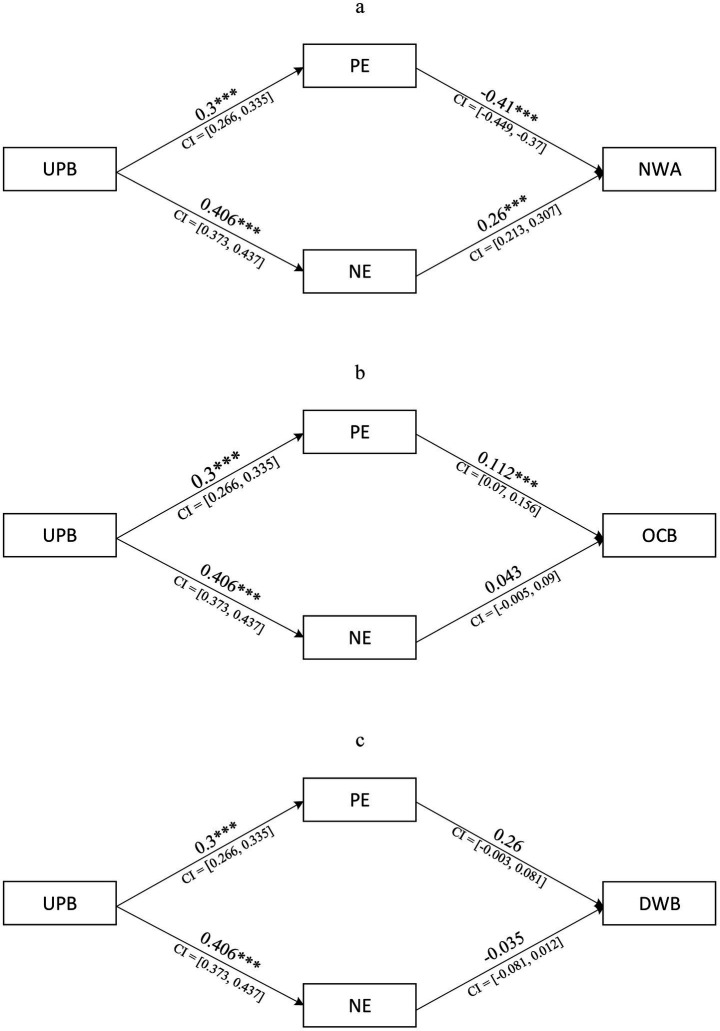
**(a)** Results of MASEM (UPB-NWA). **(b)** Results of MASEM (UPB-OCB). **(c)** Results of MASEM (UPB-DWB). *p < 0.05. **p < 0.01. ***p < 0.001.

Subsequently, the correlation matrix and corresponding standard deviations were imported into AMOS software to test the structural model, with results presented in Figure 3a (UPB-NWA), Figure 3b (UPB-OCB), and Figure 3c (UPB-DWB). The structural path results showed that UPB had a significant positive effect on positive emotion (*β* = 0.300, *p* < 0.001) and negative emotion (*β* = 0.406, *p* < 0.001). For outcome-specific paths, positive emotion exerted a significant negative effect on NWA (*β* = −0.410, *p* < 0.001), a significant positive effect on OCB (*β* = 0.112, *p* < 0.001), but no significant effect on DWB (*β* = 0.038, *p* > 0.05). Negative emotion had a significant positive effect on NWA (*β* = 0.260, *p* < 0.001) but no significant effects on OCB (*β* = 0.043, *p* > 0.05) or DWB (*β* = −0.035, *p* > 0.05). Direct effects of UPB on the three outcomes remained significant: NWA (*β* = 0.312, *p* < 0.001), OCB (*β* = 0.175, *p* < 0.001), and DWB (*β* = 0.284, *p* < 0.001).

Bootstrapping was employed to test and quantify the indirect (mediating) effects, with results summarized in Table 6. Specifically, UPB exerted a significant negative indirect effect on NWA via positive emotion (*β* = −0.123, *p* < 0.05, 95% CI excluded zero) and a significant positive indirect effect on OCB via positive emotion (*β* = 0.034, *p* < 0.05, 95% CI excluded zero), but the indirect effect of UPB on DWB via positive emotion was non-significant (*β* = 0.012, *p* > 0.05, 95% CI included zero). For negative emotion as the mediator, UPB had a significant positive indirect effect on NWA (*β* = 0.105, *p* < 0.05), while the indirect effect on OCB (*β* = 0.017, *p* > 0.05) and DWB (*β* = −0.014, *p* > 0.05) were non-significant.

**Table 6:** Results of mediation path analysis.

Path	*β*	S. E.	Z	*P*	Bootstrap 95% CI	
					Lower	Upper
Total effect						
UPB-NWA	0.294	0.02	14.700	***	0.264	0.361
UPB-OCB	0.226	0.02	11.300	***	0.123	0.227
UPB-DWB	0.282	0.019	14.842	***	0.236	0.332
Direct effect						
UPB-NWA	0.312	0.025	12.480	***	0.238	0.328
UPB-OCB	0.175	0.026	6.731	***	0.139	0.236
UPB-DWB	0.284	0.025	11.360	***	0.211	0.3
Indirect effect						
UPB-PE-NWA	−0.123	0.007	−17.571	***	−0.144	−0.104
UPB-PE-OCB	0.034	0.007	4.857	***	0.021	0.049
UPB-PE-DWB	0.012	0.007	1.714	0.07	−0.001	0.024
UPB-NE-NWA	0.105	0.012	8.750	***	0.086	0.128
UPB-NE-OCB	0.017	0.009	1.889	0.082	−0.002	0.037
UPB-NE-DWB	−0.014	0.009	−1.556	0.145	−0.034	0.005

*β*, standardized path coefficient. *p < 0.05. **p < 0.01. ***p < 0.001.

Further decomposition of the total effects clarified the nature of these mediating roles: for the UPB-NWA relationship, positive emotion exhibited a suppressor effect (*β* = −0.123, *p* < 0.05)—while UPB had a positive direct effect on NWA (*β* = 0.312), the negative indirect effect of positive emotion attenuated the total effect (*β* = 0.294, *p* < 0.05), with the adjusted direct effect accounting for 74.82% of the total effect and negative emotion exerting a partial mediating effect (*β* = 0.105, *p* < 0.05). For the UPB-OCB relationship, the direct effect (*β* = 0.175, *p* < 0.05) accounted for 77.43% of the total effect (*β* = 0.226, *p* < 0.05), with positive emotion played partial mediating roles (*β* = 0.034, *p* < 0.05) and negative emotion showing no significant mediation (*β* = 0.017, *p* > 0.05). For the UPB-DWB relationship, the direct effect (*β* = 0.284, *p* < 0.001) accounted for 93.19% of the total effect (*β* = 0.282, *p* < 0.05), with neither positive nor negative emotions demonstrating significant mediating effects (both *p* > 0.05). Collectively, these results provide partially support for hypothesis H5a (the mediating paths of positive emotion on OCB were significant, but the path on DWB was not) and partially support for hypothesis H5b (the mediating path of negative emotion on NWA was significant, but paths on OCB and DWB were not).

## 5 Discussion

As a prevalent workplace behavior, UPB has garnered growing scholarly attention, yet its implications for actors’ subsequent work attitudes and behaviors remain theoretically and empirically fragmented—particularly regarding how UPB influences OCB and DWB through emotions, for which no consensus has been reached. To address these gaps, this study employed meta-analysis to systematically examine UPB’ relationships with work attitudes (positive vs. negative), work behaviors (OCB vs. DWB), and work performance, while test sampling time, cultural background, and gender as boundary conditions. Furthermore, it explored the mediating roles of positive and negative emotions in UPB’s action mechanisms and boundary conditions, and generating evidence-based conclusions through synthetic empirical analysis.

### 5.1 Main effects of UPB on actors’ work outcomes

This study confirms that UPB elicits dual emotional responses (simultaneous positive and negative emotions) in actors, aligning with the findings of Tang et al. (2020) and Xu et al. (2024). On one hand, the pro-organizational nature of UPB leads actors to frame the behavior as a contribution to organizational interests, triggering positive emotions such as pride and belongingness. Additionally, the short-term benefits brought by UPB may also generate transient pleasure, reinforcing their self-perceptions as valuable, committed employees. On the other hand, the unethical core of UPB compels actors to recognize violations of moral norms, evoking negative emotions including guilt and shame. Compounding this, fear of behavior exposure induces anxiety, while the inherent conflict between “pro-organizational intent” and “unethical means” exacerbates psychological strain, leading to sustained unease and frustration over time.

For work attitudes, UPB was found to significantly amplify NWA (e.g., job burnout, turnover intention), validating the “moral depletion” characteristic of UPB. Employees with strong moral identity experience profound cognitive dissonance after engaging in UPB, and prolonged psychological conflict depletes their emotional resources, culminating in job burnout and prompting turnover intentions as a way to escape moral pressure (Mesdaghinia et al., 2019). In terms of work behavior, UPB exerts a “dual-promotion effect” that significantly increases both OCB and DWB—a finding consistent with Yang et al. (2022) that reflects the differentiated behavioral impacts of UPB’s dual attributes. Promotion of OCB may result from two mechanisms: first, positive emotions (e.g., pride) strengthen organizational identification, prompting employees to proactively safeguard organizational interests through helping colleagues or upholding organizational reputation. Second, negative emotions (e.g., guilt) trigger compensatory motivation, driving prosocial behaviors (e.g., improving customer service) to repair damaged moral self-concepts. Conversely, the promotion of DWB is related to UPB’s “demoralization effect”: actors may develop a sense of psychological entitlement from “sacrificing for the organization,” rationalizing self-serving behaviors (e.g., falsifying expenses); or repeatedly engaging in UPB erodes moral constraints, initiating a “what-the-hell” vicious cycle of escalating deviance (e.g., persistent deception, work withdrawal).

Contrary to the findings of Schwarz et al. (2024) and Hao et al. (2023), this study found no significant association between UPB and either positive work attitudes or work performance—a pattern attributable to the time-lag offset of short-term positive effects by long-term negative consequences. Although UPB’s pro-organizational attribute can trigger transient positive emotions in the short term, these feelings are context-dependent and transient, failing to transform into stable positive work attitudes or sustained performance improvement. Tang et al. (2020) longitudinal research confirms that UPB’s positive performance impact via pride persists for only 1–2 weeks, after which it is overshadowed by shame and guilt stemming from its unethical nature. From an attitude formation perspective, the establishment of positive work attitudes (e.g., job satisfaction, work engagement) requires long-term stable value identification and emotional experiences. However, UPB-induced moral cognitive conflict continuously consumes psychological resources, preventing short-term positive emotions from solidifying into stable attitudes. For work performance, the offsetting effect across the time dimension is more pronounced: in the short term, UPB may help achieve task goals by bypassing rules or concentrating resources (Gao, 2019), but long-term emotional exhaustion reduces work efficiency (Hao et al., 2023), while “hidden harms” such as damaged organizational reputation and lost customer trust further constrain performance growth (Xu et al., 2024). For example, employees who conceal product defects to meet short-term sales targets may temporarily boost performance, yet subsequent surges in customer complaints and declining repurchase rates offset these initial gains, ultimately resulting in no significant change in overall performance. This conclusion underscores the dynamic, time-contingent nature of UPB’s impact—short-term and partial positive effects cannot mask long-term and overall negative consequences, which ultimately manifests as non-significant association at the meta-analytic synthesis.

### 5.2 Boundary conditions of the relationships between UPB and actors’ work outcomes

This study reveals that the moderating effects of sampling time, cultural background, and gender on the UPB-work outcome relationships are complex and heterogeneous, with each moderator exerting distinct impacts across different outcome variables.

First, regarding the moderating role of sampling time, the associations between UPB and negative work attitudes, as well as between UPB and OCB, are stronger in multi-wave sampling designs than in cross-sectional data; conversely, the UPB-DWB link is more pronounced in cross-sectional data. This divergence stems from the dynamic cumulative nature of UPB’s impact on emotional and behavioral responses. On one hand, multi-wave designs better capture the long-term evolution of emotions. For example, shame stemming from UPB accumulates over time, gradually exacerbating job burnout; meanwhile, the compensatory motivation from guilt is manifested through sustained OCB. On the other hand, cross-sectional data is more likely to capture short-term impulsive responses. The immediate sense of psychological entitlement after engaging in UPB may directly trigger DWB, but this effect fades over time as moral reflection and guilt mitigate self-serving tendencies.

Second, the moderating effect of cultural background is evident. The associations between UPB and both OCB and DWB being stronger in the Eastern cultural contexts than in Western cultures. This pattern is closely tied to the collectivism orientation and Confucian-influenced moral self-discipline characteristics inherent in Eastern cultures. On one hand, Eastern collectivism internalizes “group interests overriding individual interests” as a core value, leading individuals to frame UPB’s pro-organizational attribute as a legitimate act of “sacrificing for the collective” (Chen et al., 2023). This cognition significantly enhances actors’ organizational identification and sense of belonging, amplifying their compensatory motivation. Specifically, when UPB evokes guilt, collectivist tendencies prompt individuals to link moral repair with collective interest maintenance, prompting more proactive OCB (e.g., assisting colleagues, safeguarding organizational reputation) and thus reinforcing the UPB-OCB association. On the other hand, the relationship-centric logic of Eastern collectivism weakens perceptions of UPB’s unethicality. In a highly collectivist context, actors prioritize alignment with group expectations over individual moral standards, and the moral cognitive conflict triggered by UPB is diluted by the legitimacy of “benefiting the collective” (Xu et al., 2024). This attenuated conflict reduces moral self-constraint, enabling actors to rationalize subsequent DWB through moral disengagement (e.g., “flexibility is acceptable for the team’s sake”). Meanwhile, the pressure to maintain interpersonal harmony in Eastern culture may inhibit moral dissent, blocking legitimate channels for releasing UPB-induced negative emotions. Ultimately, these emotions then manifest as DWB (e.g., work withdrawal and interpersonal alienation), further strengthening the UPB-DWB linkage.

Third, the moderating effect of gender is noteworthy. The higher the proportion of females, the weaker the association between UPB and DWB. This core finding stems from female heightened moral sensitivity and distinct emotion regulation patterns. On one hand, the process of gender socialization leads women to develop a stronger role identity as “moral guardians” (Eagly and Sczesny, 2019) and a lower perceptual threshold for moral violations. After engaging in UPB, women are more likely to experience intense guilt and shame due to the behavior’s “unethical attribute.” These self-conscious emotions activate the motivation for “moral self-repair,” prompting women to atone for their mistakes through compliant behaviors (e.g., taking the initiative to assume additional work, improving service quality) rather than releasing pressure through DWB (Khawaja et al., 2023). In contrast, men are more prone to rationalizing UPB through “legitimacy of purpose,” experience weaker moral emotions, and are more inclined to balance the psychological cost of “sacrificing for the organization” through self-serving DWB. On the other hand, women’s emotion regulation patterns tend to be “active adaptation” rather than “passive venting.” The moral conflict triggered by UPB can cause women to experience stronger psychological distress, but they are more likely to alleviate negative emotions through interpersonal communication, self-reflection, and other means, rather than engaging in DWB that violates organizational norms (Gigol, 2021). Additionally, women attach greater importance to interpersonal relationships and worry that DWB will undermine workplace harmony; this “relationship maintenance” motivation further inhibits the transformation of UPB into DWB. The non-significant moderating effect of gender on the relationship between UPB and OCB may reflect the convergence of gender roles in social development. Regardless of gender, actors may seek moral balance through OCB under the compensatory motivation triggered by UPB. This finding breaks the conventional perception that “gender differences inevitably lead to behavioral differentiation” and enriches the connotation of demographic variables in UPB research.

### 5.3 Mediating mechanisms of the relationships between UPB and actors’ work outcomes

Based on AET, this study reveals the differentiated mediating roles of positive and negative emotions in the relationships between UPB and work outcomes.

This study indicates that UPB exerts a negatively influences on negative work attitudes through positive emotions, while it positively influences such attitudes through negative emotions. This “dual-offset mechanism” reflects the emotional paradox of UPB. Positive emotions alleviate emotional exhaustion by strengthening self-worth, weakening turnover intentions, and to some extent masking the positive direct effect of UPB on negative work attitudes. Negative emotions deplete psychological resources by exacerbating cognitive dissonance, directly increasing job burnout and turnover intentions, and serving as the core transmission path for UPB’s negative impacts.

Both positive and negative emotions play positive mediating roles in the relationship between UPB and OCB, confirming the dual logic of “emotion-driven behavioral compensation.” According to the Broaden-and-Build Theory of Positive Emotions (Fredrickson, 2001), positive emotions expand cognitive scope and enhance social connections, prompting employees to proactively participate in organizational activities (e.g., helping colleagues, offering suggestions) to maintain a positive self-image. From the perspective of Emotion Feedback Theory (Baumeister et al., 2007), negative emotions act as “moral warnings,” driving employees to reduce future guilt through OCB (e.g., improving customer service) and achieve moral self-repair.

Positive emotions positively mediate the relationship between UPB and DWB, while the mediating role of negative emotions is non-significant. This result reveals a unique emotional path through which UPB drives DWB, differing from the conventional logic in existing studies that negative emotions dominate DWB. The specific mechanisms can be explained from two aspects. On one hand, Positive emotions triggered by UPB may promote DWB through the “moral licensing effect.” When actors experience pride due to the “pro-organizational” nature of UPB, they may interpret it as “special contributions to the organization,” thereby developing a sense of psychological entitlement (i.e., “I deserve extra rewards”). This sense of entitlement weakens moral self-constraint, enabling actors to rationalize subsequent self-serving behaviors (e.g., falsifying expenses, slacking off)—even perceiving them as “reasonable claims from the organization.” For example, an employee who feels proud of “securing an order by concealing information” may believe that “occasional lateness or perfunctory work is well-deserved compensation.” Additionally, the cognitive broadening effect of positive emotions (Fredrickson, 2001) may make actors more likely to find “legitimate reasons” for DWB (e.g., “everyone does this”), further reducing guilt about deviant behaviors. On the other hand, the non-significant mediating role of negative emotions may stem from the “neutralization effect” of UPB’s “pro-organizational nature” on negative emotions. Actors may attribute negative emotions to “sacrifices made for collective interests” rather than purely “personal moral failures,” thereby weakening the inhibitory effect of negative emotions on behavior. For example, an employee may feel guilty about deceiving customers but rationalize this emotion by thinking “this is for the company’s survival,” preventing guilt from translating into self-restraint against DWB.

Furthermore, if the organization implicitly condones UPB, actors may perceive negative emotions as “excessive sensitivity,” further weakening their impact on subsequent behaviors. This finding suggests that UPB drives DWB not through the outburst of negative emotions from the effect of “what-the-hell,” but rather primarily through the “expansion of entitlement” and “loosening of moral standards” triggered by positive emotions. It provides a new emotional perspective for understanding the association between UPB and deviant behaviors.

## 6 Research contributions, limitations, and future directions

### 6.1 Theoretical contributions

First, this study reveals the unique mechanism through which UPB influences DWB through positive emotions, challenging the traditional view that negative emotions are the primary drivers of deviant behavior. Previous studies have mostly argued that negative emotions triggered by unethical behavior are the core drivers of DWB. However, this study finds that UPB positively influences DWB through positive emotions, and this path is independent of negative emotions. This conclusion validates the “moral licensing effect”: positive emotions may lead actors to develop a sense of psychological entitlement (i.e., “deserving privileges due to contributions”), thereby rationalizing subsequent deviant behaviors. It provides a new emotional perspective for understanding the association between UPB and DWB and enriches the application of AET in explaining the “double-edged sword effect of positive emotions.”

Second, this study clarifies the differentiated mediating roles of emotions in the relationship between UPB and work behaviors, improving the dynamic impact model of UPB. Specifically, positive emotions simultaneously drive OCB (via compensatory motivation) and DWB (via inflated sense of entitlement), whereas negative emotions only significantly mediate the positive impact of UPB on OCB (via moral repair) and exert no significant mediating influence on DWB. This finding dispels that emotions exert unidirectional behavior effects, demonstrating that UPB-triggered emotional responses can diverge into constructive and destructive behaviors through distinct psychological mechanisms. It provides a more nuanced theoretical framework for resolving the “behavioral paradox” of UPB.

Third, this study systematically synthesizes fragmented findings on UPB’s consequences via meta-analysis, generating robust benchmark evidence for future inquiry. It confirms that UPB exerts a significant positive impact on negative work attitudes, OCB, and DWB, while clarifying the pivotal role of positive emotions in the UPB-DWB pathway. This addresses inconsistencies in prior empirical results (e.g., conflicting claims that UPB only promotes OCB or only drives DWB) and lays a foundation for cross-contextual comparisons and mechanism refinement of UPB’s outcomes.

Fourth, this study verifies that sampling methods, cultural background, and gender exhibit different moderating effects (in both direction and magnitude) on UPB-work outcomes relationship. These conclusions enrich the content and hierarchy of boundary conditions for UPB, advancing and integrating existing research on the contingencies of UPB’s impacts.

### 6.2 Practical implications

First, managers should embed ethical guidelines into performance appraisal systems, improve the time dimension of performance evaluation by combining short-term performance with long-term compliance, reputation indicators, and other metrics, and establish a performance retrospective assessment period. For short-term performance gains achieved through UPB, if subsequent issues such as customer complaints or reputational damage arise, the relevant performance rewards should be retroactively deducted to reduce the attractiveness of UPB’s fleeting benefits. In addition, firms should establish an early-warning and remediation mechanism for UPB consequences. The human resources (HR) and ethics departments should jointly develop “Classification Handling Guidelines for UPB Behaviors.” For minor UPB (e.g., mild exaggeration of publicity to protect team image), employees should be guided to conduct moral repair through OCB, such as proactively clarifying facts or enhancing service quality. For severe UPB (e.g., irregular operations causing organizational losses), formal compliance accountability and reputation repair procedures should be initiated to prevent short-term gains from overshadowing long-term negative consequences.

Second, to address the emotional contradictions triggered by UPB, organization should implement differentiated emotion management strategies combining short-term interventions and long-term support. For example, after the occurrence of UPB, one-on-one emotional counseling should be promptly deployed to guide employees in expressing negative emotions, preventing emotional suppression from escalating into DWB or emotional exhaustion. In addition, long-term, regular psychological counseling should be provided for roles with high UPB exposure risk (e.g., managerial positions, core business roles) to strengthen employees’ psychological resource stock, enhance their capacity to navigate moral conflicts, and mitigate performance decline and attitude deterioration stemming from emotional depletion.

Third, management strategies should be tailored to cultural backgrounds and gender differences. In the Eastern cultural context, HR and ethics managers must balance the advantages of collectivism with clear ethical boundaries. Through ethics training (e.g., case-based teaching including positive cases and negative cases), employees should be guided to recognize that “collective interests at the expense of morality are not true collective values,” preventing UPB from being rationalized as “sacrifice for the collective.” To address the issue of “interpersonal harmony pressure inhibiting moral dissent” in collectivist culture, an anonymous online feedback platform should be established to enable employees to report or comment on UPB-related behaviors, reducing the interpersonal risks of moral expression and preventing the accumulation of negative emotions from manifesting as DWB. Considering the mechanism by which a higher proportion of females weakens the UPB-DWB association, HR can optimize team configuration in high-risk scenarios by increasing the proportion of female employees in UPB-prone roles (e.g., sales, procurement, where behaviors such as “concealing defects for performance” or “irregular operations for cooperation” are likely to occur) to leverage their high moral sensitivity and relationship maintenance motivation, thereby inhibiting the spread of UPB to DWB at the team level.

### 6.3 Research limitations and future directions

Despite yielding valuable conclusions and theoretical/practical insights, this study has several limitations that warrant further refinement.

First, the subdivision of positive emotions is insufficient. This study treats positive emotions as an aggregate variable, failing to distinguish between specific types (e.g., pride, pleasure, belongingness). In fact, different positive emotions may operate through distinct mechanisms, for example, pride is more likely to trigger psychological entitlement, whereas pleasure may strengthen social connectedness. A similar limitation applies to the aggregation of negative emotion. Future research should further subdivide positive emotions to uncover more precise mediating pathways.

Second, the meta-analysis for certain variables is constrained by the nascent stage of UPB consequences research. For instance, the association between positive emotions and DWB is based on only 5 studies, potentially compromising the robustness of related conclusions. Additionally, heterogeneity may stem from divergent measurement approaches for positive emotions across studies (e.g., self-report vs. physiological indicators). Future research should prioritize accumulating empirical studies to enable more comprehensive and reliable analysis of UPB’s impacts on work outcomes.

Third, while meta-analysis can integrate existing conclusions, it cannot verify causal relationships. Future studies could adopt experimental designs or longitudinal tracking to manipulate UPB contexts and dynamically monitor changes in emotions and behaviors, thereby enhancing the causal inference of conclusions.

Fourth, this study only tests the moderating effects of sampling time, cultural background, and gender and does not involve. Future research should further explore the moderating effects more aligned with AET, such as organizational-level variables (e.g., ethical climate, leadership style, power distance) or individual-level variables (e.g., moral identity, narcissism). Exploring these moderating variables from the perspective of AET can further enrich the boundary conditions of the influence of UPB.

Fifth, although this study adopts a meta-analytic design, integrating independent data from different samples and research contexts, which to a certain extent reduces the impact of Common-Method Variance (CMV) in single studies, it is necessary to acknowledge potential limitations. Some of the included original studies may use a cross-sectional design and rely on data collection from the same source (e.g., employees’ self-reported questionnaires measuring UPB, emotional variables, and outcome variables simultaneously). Such research designs themselves are difficult to completely rule out the interference of CMV, which may lead to a slight overestimation of the correlation coefficients between variables (Podsakoff et al., 2012). In addition, since some original studies did not explicitly report the results of CMV tests (such as Harman’s single-factor test and confirmatory factor analysis), we cannot further quantify or control this potential bias at the meta-analytic level. Future studies may prioritize the use of multi-source data collection (e.g., employees’ self-assessment of UPB, colleagues’ evaluation of performance, supervisors’ evaluation of behavior) or multi-time-point tracking designs to more directly avoid the impact of CMV.

In summary, this study provides a systematic perspective for understanding the complex consequences of UPB. However, as a form of “morally gray behavior,” UPB’s impact mechanisms demand more refined research to provide more precise theoretical guidance for organizational ethical management.

## Data Availability

The original contributions presented in the study are included in the article/supplementary material, further inquiries can be directed to the corresponding author.
